# The surgical pathology laboratory in Mwanza, Tanzania: a survey on the reproducibility of diagnoses after the first years of autonomous activity

**DOI:** 10.1186/s13027-017-0115-z

**Published:** 2017-01-21

**Authors:** R. Tumino, P. F. Rambau, F. Callea, L. Leoncini, R. Monaco, J. Kahima, V. Stracca Pansa, L. Viberti, D. Amadori, P. Giovenali, K. A. Mteta

**Affiliations:** 1 Cancer Registry and Histopathology Department, “Civic – M.P. Arezzo” Hospital, ASP Ragusa, Ragusa, Italy; 2 Patologi Oltre Frontiera (Pathologists beyond borders) NGO, Milan, Italy; 3 Bugando Medical Centre, Pathology Department, Mwanza, Tanzania; 4 Department of Pathology, “Ospedale Pediatrico Bambin Gesù” Children’s Hospital, Rome, Italy; 5 Department of Human Pathology and Oncology, University of Siena, Siena, Italy; 6 Anatomical Pathology Unit, AORN Cardarelli, Naples, Italy; 7 Sedes Sapientae Hospital, Turin, Italy; 8 Romagna Scientific Institute for Cancer Study and Cure (IRST) - IRCCS, Meldola, Italy; 9 Department of Pathology “S. Maria della Misericordia” Hospital, Perugia, Italy

**Keywords:** Reproducibility, K-statistic, Error, Quality control, Low-Human Development Index countries, Pathology diagnosis

## Abstract

**Background:**

In 2000, an Italian non-governmental organisation (NGO) began a 9-year project to establish a surgical pathology laboratory at the Bugando Medical Centre (BMC) in Mwanza, Tanzania, a country with a low Human Development Index (HDI), and as of 2009, the laboratory was operating autonomously. The present survey aims to evaluate the reproducibility of histological and cytological diagnoses assigned in the laboratory’s early years of autonomous activity. We selected a random sample of 196 histological and cytological diagnoses issued in 2010–2011 at the BMC surgical pathology laboratory. The corresponding samples were sent to Italy for review by Italian senior pathologists, who were blinded to the local results. Samples were classified into four diagnostic categories: malignant, benign, inflammatory, and suspicious. The two-observer kappa-statistic for categorised (qualitative) data was then calculated to measure diagnostic concordance between the local Tanzanian pathologists and Italian senior pathologists. The k-Cohen was calculated for concordance in the overall study sample. Concordance and discordance rates were also stratified by subset: general adult, paediatric/adolescent, and lymphoproliferative histopathological diagnoses; fluid and fine needle aspiration (FNA) cytological diagnoses; and PAP tests. Discordance was also categorised by the corresponding hypothetical clinical implications: high, intermediate, and not significant.

**Results:**

Overall concordance was 85.2% (167 of 196 diagnoses), with a k-Cohen of 0.7691 (*P* = 0.0000). Very high concordance was observed in the subsets of adult general pathological diagnoses (90%) and paediatric/adolescent pathological diagnoses (91.18%). Concordance in the subset of PAP tests was 75%, and for fluid/FNA cytological diagnoses it was 56.52%. Concordance among 12 histological subtypes of lymphoma was 75.86%, with substantial discordance observed in the diagnosis of Burkitt lymphoma (five cases diagnosed by Italian pathologists versus 2 by local pathologists). The overall proportion of discordance with high hypothetical clinical implications was 6.1% (12 diagnoses).

**Conclusion:**

This blind review of diagnoses assigned in Tanzania, a country with low HDI, and in Italy, a country with a very high HDI, seemed to be a sensitive and effective method to identify areas of potential error and may represent a reference point for future, more detailed quality control processes or audits of surgical pathology services located in limited-resource regions.

## Background

The United Republic of Tanzania is located in East Africa and has a low Human Development Index (HDI), ranking 159^th^ out of the 187 countries classified by the United Nations Development Programme [24]. The Bugando Medical Centre (BMC) is one of four main referral hospitals in Tanzania. It is a 900-bed facility that also serves as a consulting and teaching hospital for the Catholic University of Health and Allied Sciences. Located in Mwanza, the second largest city in Tanzania, the BMC employs approximately 950 people and serves a catchment area of approximately 13 million inhabitants in the northwestern “Lake Zone”, i.e., one-third of the country’s total population of about 40 million.

In 2000, a 9-year project was started, the Mwanza histopathology project, to establish a surgical pathology laboratory at the BMC. This project was funded by the Italian charity “Vittorio Tison Association” (http://www.associazionevittoriotison.org) and carried out by the Italian non-governmental organisation (NGO) “Pathologists beyond borders” (Associazione Patologi Oltre Frontiera; http://www.apof.eu/). The project included the training of local staff (two pathologists, one histotechnologist, and one lab technician), and the continued presence of Italian senior pathologists, histotechnologists, and biologists through monthly rotations. Their role was to run the histopathology activities while training and supervising the local pathologists and lab technicians. Their presence ended at the end of 2008, and as of 2009 the laboratory was operating autonomously.

Currently, the BMC surgical pathology laboratory renders about 3,500 histological and 800 cytological diagnoses per year. This represents a unique reality in this sub-Saharan African region, where the physician-to-population ratio is one of the lowest in the world (1:23,000), and there are just 15 pathologists in the entire nation [17]. But practicing pathology in this resource-limited setting is a strenuous challenge [17] and may affect the quality of diagnoses. Furthermore, evaluating the performance of professionals is a core necessity in any health system and has been the object of much debate in the literature, especially in this post-genome era. This has generated more demands on pathology departments, as well as a commitment from pathologists to strive for the highest quality possible in their laboratories [5, 9, 11, 19].

Finally, as in many other sub-Saharan African countries, the cancer burden in Tanzania is increasing [1]. The most common female malignancy is cervical cancer, and childhood malignancies are also quite common, including lymphomas like Burkitt lymphoma (BL). In the early 1990s, female breast cancer represented about 8.1% of all cancers in women, but it is now on the rise, and women present at a younger age than in Western countries [2, 17]. Non-Hodgkin lymphoma, Kaposi sarcoma, foot melanoma, and cancers of the bladder, esophagus, stomach, and prostate are the most common tumours in men [7, 14, 17]. Given this rise in disease, the BMC surgical pathology laboratory has the potential to play a key role in providing cancer statistics, and to contributing to secondary cancer prevention and cancer control nationwide. However, to accomplish this, the laboratory must adhere to the best quality standards possible, with a special emphasis on diagnostic issues.

For this reason, this survey evaluated the reproducibility of histological and cytological diagnoses assigned in the early years of autonomous activity (2010–2011) of the BMC surgical pathology laboratory, with the aim to assess the capability of local staff to provide histopathological and cytopathological services at a reasonable level of quality in a limited-resource country.

## Methods

The present survey is based on a sample representing 5% of the average number of diagnoses assigned annually at the BMC surgical pathology laboratory. To achieve this, we selected one of every 45 consecutive histological reports and one of every 40 consecutive cytological reports issued from July 2010 (after the approval of the collaboration between the BMC surgical pathology laboratory and Italian organisations from the Tanzanian Ministry of Health and Social Welfare) to June 2011 from the laboratory’s registry book, until we reached 172 histological and 43 cytological reports. Reports were selected in such a way that every calendar month was represented. Reports were then categorised into the following subsets: general adult pathological diagnoses, paediatric/adolescent pathological diagnoses (age 0–19 years), lymphoproliferative pathological diagnoses, fluid and fine needle aspiration (FNA) cytological diagnoses, and PAP tests.

Corresponding samples were then sent from the BMC surgical pathology laboratory to Italy for review by Italian senior pathologists (chiefs of pathology departments of public Italian hospitals and universities). These included haematoxylin-eosin slides, histology blocks to determine the subtype of lymphoproliferative pathologies by immunohistochemistry (as appropriate), and papanicolau-stained fluid/FNA cytological slides and cervical smears. The Italian reviewers were blinded to the local diagnosis assigned by their Tanzanian counterparts, but they were given all available clinical history, as well as the type of biopsy and the site from which it was taken. The Italian reviewers assigned their own diagnoses, which were then classified into the same four diagnostic categories used by local pathologists, i.e., malignant, benign, inflammatory, and suspicious (cervical intraepithelial neoplasia grades 1 and 2 were categorised as malignant and atypical squamous cells of undetermined significance as suspicious).

We calculated the two-observer kappa-statistic for categorised (qualitative) data in the overall study sample to measure concordance between diagnoses assigned by local pathologists and Italian senior pathologists according to Cohen [4] and Fleiss [8]. The standard error of k-statistics for the overall sample was computed under the z statistic. The k value was ranked according to Landis and Kock [13] as poor (0 – 0.2), fair (0.21 – 0.4), moderate (0.41 – 0.6), substantial (0.61 – 0.8), or almost perfect (>0.8). We then calculated concordance and discordance rates stratified by subset. Concordance among lymphoproliferative pathological diagnoses was also computed for the 12 subtypes of lymphoma. Finally, discordance was also categorised by the corresponding hypothetical clinical implications: high (malignant versus benign; any cervical intraepithelial neoplasia versus benign/inflammatory for PAP tests); intermediate (suspicious and atypical squamous cells of undetermined significance versus benign/inflammatory; differences in subtypes of lymphoma); and not significant (benign versus inflammatory). The statistical package STATA 10.0 was used for the analysis.

## Results

Our study sample was based on a final number of 196 diagnoses, because 19 slides were damaged or misplaced during transport to Italy. The final samples corresponded to 90 general adult pathological diagnoses, 34 paediatric/adolescent pathological diagnoses, 29 lymphoproliferative pathological diagnoses, 23 fluid/FNA cytological diagnoses and 20 PAP tests. The original diagnoses from the BMC surgical pathology laboratory were related to the following anatomical sites: cervix uteri (42, including PAP tests, 21.4%), lymph nodes (29, 14.8%), female breast (17, 8.7%), skin (16, 8.2%), prostate and intraabdominal organs (12 each, 6.1% each), ovary (11, 5.6%), small and large bowel (7, 3.6%), thyroid and bone (6 each, 3.1% each), kidney (5, 2.5%), submandibular tissue (4, 2.1%), oesophagus (3, 1.5%), and testis (2, 1%). Apart from PAP tests, there were more samples from females than males (109 and 63 samples, respectively), and most of the patients were 20 to 50 years of age (accounting for 80 of 196 cases, 40.8%); 56 cases were under 20 years of age (28.6%) (Table 1).

**Table 1:** Anatomical site of samples received from the BMC, and sex and age of the study sample by subset

Anatomical site	Subset					
	General adult pathological diagnoses	Paediatric/adolescent pathological diagnoses	Lymphoproliferative pathological diagnoses	Fluid/FNA cytological diagnoses	PAP test	Total
Cervix	21	1	-	-	20	42 (21.4%)
Lymphnode	-	-	22	7	-	29 (14.8%)
Breast female	6	4	-	7	-	17 (8.7%)
Skin	11	5	-	-	-	16 (8.2%)
Prostate	12	-	-	-	-	12 (6.1%)
Intrabdominal	6	1	-	5	-	12 (6.1%)
Ovary	6	2	3	-	-	11 (5.6%)
Small/Large bowel	5	2	-	-	-	7 (3.6%)
Thyroid	5	-	-	1	-	6 (3.1%)
Bone	-	6	-	-	-	6 (3.1%)
Kidney	2	3	-	-	-	5 (2.5%)
Submandibular	3	1	-	-	-	4 (2.1%)
Oesophagus	3	-	-	-	-	3 (1.5%)
Testis	2	-	-	-	-	2 (1.0%)
Other	5	9	4	3	-	21 (10.7%)
Total	90	34	29	23	20	196 (100%)
Sex						
F	57	21	16	15	20	129 (65.8%)
M	33	12	10	8	-	63 (32.2%)
Unknown	-	1	3	-	-	4 (2.0%)
Total	90	34	29	23	20	196 (100%)
Age (years)						
0–19	0	34	14	7	1	56 (28.6%)
20–50	54	-	9	7	10	80 (40.8%)
51–74	24	-	1	7	2	34 (17.4%)
75+	8	-	2	2	0	10 (5.1%)
Unknown	4	-	5	0	7	16 (8.2%)
Total	90	34	29	23	20	196 (100%)

*FNA* fine needle aspiration

Concordance in the overall study sample was 85.20% (k-Cohen 0.7691, *P* = 0.0000), with concordance in 167 of 196 diagnoses (77 malignant, 67 benign, 21 inflammatory, and two suspicious). Discordance occurred in six samples classified as malignant by Italian reviewers but reported as benign (*n* = 5) or inflammatory (*n* = 1) by BMC pathologists, three samples classified as benign by Italian reviewers that were reported as malignant by BMC pathologists, and three samples classified as inflammatory according to Italian reviewers that were reported as malignant by BMC pathologists (Table 2).

**Table 2:** Overall diagnostic concordance by the four diagnostic categories considered

BMC – MWANZA	Agreement	Kappa	St. error	Prob > z	
	85.20%	0.7691	0.0505	0.0000	
	Italian reviewer				
	Malignant	Benign	Inflammatory	Suspicious	Total
Malignant	77	3	3	3	86
Benign	5	67	3	0	75
Inflammatory	1	6	21	4	32
Suspicious	0	1	0	2	3
Total	83	77	27	9	196

*BMC* Bugando Medical Centre

In the subset of lymphoproliferative pathological diagnoses (*n* = 29), concordance was 93.1% when the analysis was based on the four main diagnostic categories. In fact, there was a disagreement for malignancy in only two instances: one Italian-diagnosed non-Hodgkin unclassifiable lymphoma versus BMC-diagnosed inflammatory; and one Italian-diagnosed T-Cell lymphoma versus BMC-diagnosed thymoma. However, when we looked at the agreement by histological subtype of lymphoma, the concordance decreased to 75.86% (Table 3). Discordance was observed in the diagnosis of BL (five cases diagnosed by Italian reviewers versus two at the BMC; the remaining BMC diagnoses included two diffuse large B-cell lymphoma; and one lymphoblastic lymphoma) and diffuse large B-cell lymphoma (five cases diagnosed by Italian reviewers versus eight at the BMC).

**Table 3:** Diagnostic concordance in lymphoproliferative pathological diagnoses by histological subtype of lymphoma

BMC-MWANZA	Concordance							Discordance					
	75.86%							24.14%					
	Italian reviewer												
	BL	DLBCL	FL	HL	INFL	KAPOSI	LG	LBL	NHL-NOS	PBL	TCL	THYMOMA	TOTAL
BURKITT	2	0	0	0	0	0	0	0	0	0	0	0	2
DLBCL	2	5	0	0	0	0	0	0	0	1	0	0	8
FOLLICULAR	0	0	2	0	0	0	0	0	0	0	0	0	2
INFLAMMATORY	0	0	0	0	7	0	0	0	0	0	0	0	7
HODGKIN LYMPH.	0	0	0	3	0	0	1	0	0	0	0	0	4
KAPOSI	0	0	0	0	0	1	0	0	0	0	0	0	1
LYMPHOMATOID GR.	0	0	0	0	0	0	0	0	0	0	0	0	0
LYMPHOBLASTIC	1	0	0	0	0	0	0	1	0	0	0	0	2
NHL-NOS	0	0	0	0	1	0	0	0	1	0	0	0	2
PLASMABLASTIC	0	0	0	0	0	0	0	0	0	0	0	0	0
T-CELL LYMPH.	0	0	0	0	0	0	0	0	0	0	0	0	0
THYMOMA	0	0	0	0	0	0	0	0	0	0	1	0	1
TOTAL	5	5	2	3	8	1	1	1	1	1	1	0	29

*BL* BURKITT LYMPHOMA, *BMC* Bugando Medical Centre, *DLBCL* DIFFUSE LARGE B-CELL LYMPHOMA, *FL* FOLLICULAR LYMPHOMA, *HL* HODGKIN LYMPHOMA, *INFL* INFLAMMATORY, *LG* LYMPHOMATOID GRANULOMATOSIS, *NHL-NOS* NOH HODGKIN LYMPHOMA UNCLASSIFIABLE, *PBL* PLSMABLASTIC LYMPHOMA, *TCL* T-CELL LYMHOMA

There was 90% concordance in the subset of 90 adult general pathological diagnoses when the analysis was based on the four main diagnostic categories (Table 4). However, when considering the specific histological subtypes of these diagnoses the concordance decreased slightly to 87.80% (detailed data not shown). A high concordance (91.18%) was also observed in the subset of 34 paediatric/adolescence pathological diagnoses when the analysis was based on the four main diagnostic categories, but when the specific histological subtypes of these diagnoses were considered, the concordance decreased to 76.47% (detailed data not shown). For the 23 fluid/FNA cytological diagnoses, the concordance was 56.52%. Finally, the concordance in the 20 PAP tests was 75% (Table 4).

**Table 4:** Diagnostic concordance by the four diagnostic categories and by the histological subtypes of these diagnoses

SUBSET	Number	Concordance by diagnostic categories (^a^)	Concordance by histological subtype (^b^)
General adult pathological diagnoses	90	81 (90.00%)	79 (87.80%)
Paediatric/adolescent pathological diagnoses	34	31 (91.18%)	26 (76.47%)
Lymphoproliferative pathological diagnoses	29	27 (93.10%)	22 (75.86%)
Fluid/FNA cytological diagnoses	23	13 (56.52%)	-
PAP tests	20	15 (75.00%)	-

*FNA* fine needle aspiration

(^a^) = on the basis of malignant/benign/inflammatory/suspicious categories

(^b^) = on the basis of specific histo-types

Table 5 shows that, in order of decreasing magnitude, diagnostic discordance with high hypothetical clinical implications accounted for 13.0% (three of 23 diagnoses) of the subset of fluid/FNA cytological diagnoses, 6.9% (two of 29 diagnoses) of the lymphoproliferative pathological diagnoses, 5% (one of 20 diagnoses) of the PAP tests, 2.9% (one of 34 diagnoses) of paediatric/adolescent pathological diagnoses, and 2.5% (five of 90 diagnoses) of the general adult pathological diagnoses. Overall, high hypothetical clinical implications were found in 12 diagnoses (6.1%). Discordance with intermediate hypothetical clinical implications was found in 12 cases: five (21.7%) fluid/FNA cytological diagnoses, four lymphoproliferative subtype histological diagnoses (13.8%), two (2.2%) general adult pathological diagnoses and one (5%) PAP test. Discordance with no clinical significance was found in ten cases: three PAP tests (15%), two fluid/FNA cytological diagnoses (8.7%), two general adult pathological diagnoses (2.2%), two paediatric/adolescent pathological diagnoses (5.9%) and one lymphoproliferative pathological diagnosis (3.4%) (Table 5).

**Table 5:** Diagnostic discordance in the subsets by the four diagnostic categories and hypothetical clinical implications

SUBSET	Number	Discordance by four categories(^a^)	High	Intermediate	Not significant
General adult pathology	90	9 (10.0%)	5(5.6%)	2 (2.2%)	2 (2.2%)
Paediatric/adolescence	34	3 (8.8%)	1 (2.9%)	0	2 (5.8%)
Lymphoproliferative	29	2 (6.9%)	2 (6.9%)	-	-
Fluid/FNA cytological diagnoses	23	10 (43.5%)	3(13.0%)	5 (21.7%)	2 (8.7%)
PAP test	20	5 (25.0%)	1 (5.0%)	1 (5.0%)	3(15.0%)
Overall	196	29 (14.8%)	12 6.1%)	8 (4.1%)	9 (3.6%)
Lymphoproliferative by histological subtype (^b^)	29	7 (24.1%)	2 (6.9%)	4 (13.8%)	1 (3.4%)

*FNA* fine needle aspiration

(^a^) = on the basis of malignant/benign/inflammatory/suspicious categories

(^b^) = on the basis of 12 histo-types

**Fig. 1:**
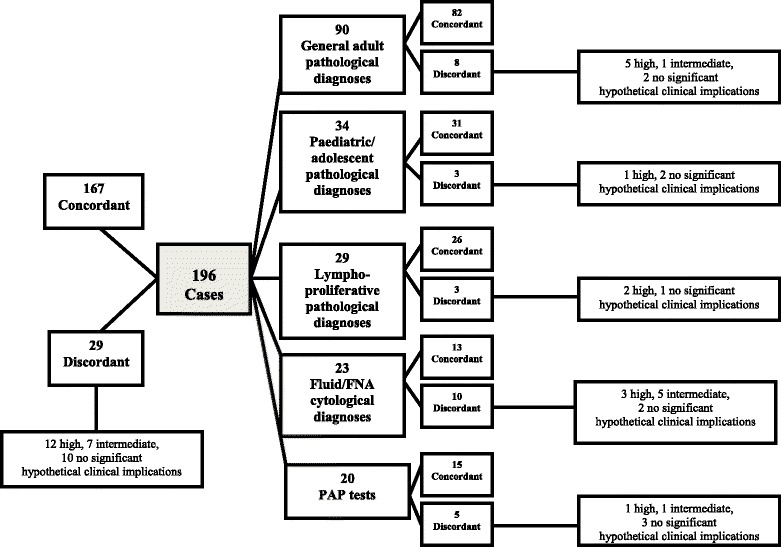
Breakdown of the 196 diagnoses by subset, concordance and discordance (*), and hypothetical clinical implications. (*): Discordance on the basis of malignant/benign/inflammatory/suspicious classification.

Figure 1 depicts the breakdown of cases by subset, indicating number of discordant diagnoses and their clinical significance.

## Discussion

To the best of our knowledge, this work is the first on the reproducibility of histological and cytological diagnoses from a hospital located in a low-HDI country where a surgical pathology laboratory has been operating autonomously for just over 2 years. Furthermore, this review is inter-departmental, inter-continental, independent, and blinded, which makes it different from many others studies that used an intra-departmental approach [9, 22]. Similarly, but not equally, to the error definition by Renshaw [18], we looked at the hypothetical clinical implications of discordance, and separated them into high hypothetical clinical implications (those in which treatment or prognosis might be different and likelihood of significantly altering patient investigation and treatment), intermediate hypothetical clinical implications (those in which the second opinion was not definitive to modify clinical management substantially), and no significant hypothetical clinical implications (those that did not imply different or relevant approaches to clinical management).

The overall findings showed a substantial concordance between external reviewers from a country with very high HDI and local pathologists from a country with a low HDI, and this concordance was very high in adult general and paediatric/adolescent pathological diagnoses. This indicates that, for the most common pathologies that exist in the Lake Region of Tanzania, there is a very high chance of receiving a reliable diagnosis based on the four diagnostic categories we considered (malignant, benign, inflammatory, or suspicious). We observed discordance with high hypothetical clinical implications in only 6.1% of diagnoses. Moreover, concordance was quite high even for lymphoproliferative diagnoses and for the histological subtypes of lymphoma. This is surprising because the original diagnoses were not supported by immunohistochemistry, which is not available at the local level. However, this may be one reason for the considerable discordance in one case of benign thymoma diagnosed at the BMC, which was diagnosed as a T-cell lymphoma in the Italian review, as well as one case of non-Hodgkin lymphoma and three cases of BL that were missed by BMC pathologists. It is worth mentioning that a standard diagnosis of BL should always be supported by the recommended algorithmic approach of Naresh et al. [15]. This discordance might be associated to a 6.9% of error with high hypothetical clinical implications. The few instances of discordance we observed in general adult pathological diagnoses were not related to the lack of immunohistochemistry. For example, one case of focal prostatic adenocarcinoma diagnosed by BMC pathologists was not mentioned by the Italian reviewer. In our opinion, these pathologists could come to a consensus on this and other cases of discordance on suspicious cases if a consensus conference were possible. To a lesser extent, the discordance observed in PAP tests could be related to the absence of screening programmes at the time of this survey. Moreover, discordance was not related to the misclassification of invasive cervical cancer (in fact it refers to low-grade squamous intraepithelial lesions), and finally, seven and six of 20 smears were defined as technically insufficient or satisfactory with limitations, respectively, by the Italian reviewer.

Technical issues may also be responsible for the worst results and for the high hypothetically clinically significant implications found in the subset of fluid/FNA cytological diagnoses: in fact, none of the cytological slides were considered technically satisfactory by the Italian reviewer, who considered them insufficient or just sufficient for staining, and/or thickness, and/or cellular representativeness of smear. This could be related to the main problems facing the practice of pathology in Tanzania reported by Ngoma and Diwani [16] and Stefan et al. [23], i.e., a low number of histopathology technicians, poor recruitment of these technicians, and difficulty in obtaining and replacing essential reagents.

This analysis has some limitations: it is not a comprehensive evaluation of errors in surgical pathology according to Renshaw [18], because we did not proceed to the second step, which involves revision of discordant diagnoses by the original pathologist and the second reviewer together, and an eventual third step (submission of cases to a third, outside reviewer as a “gold standard” in the event of lack of consensus of the first two observers) in order to classify definitive diagnostic errors. The sample was probably not large enough to detect a reasonable percentage of errors according to Renshaw et al. [22], and finally, we did not assess discordance by other, more detailed, important correlates like grade of differentiation in tumours or specific morphological prognostic characteristics [9].

With regard to the sample size, we assumed that the initial number of 215 diagnoses was appropriate and would represent the best available data on the performance of a surgical pathology laboratory operating in sub-Saharan Africa. According to Cantor [3], the final overall sample of 196 cases seems reasonable to detect an inter-rater agreement in the range of 30 to 50%, with an error margin of 20%, and a kappa of 0.75 against a null hypothesis of a kappa of 0.6 (i.e., that the agreement is substantial, not moderate), which would have required 199 assessments made by two observers to achieve 90% power. Furthermore, as this was intended to be an exploratory survey, we did not evaluate discordance by grade of differentiation in tumours or specific morphological prognostic characteristics.

The rather constrained nature of our diagnostic categories (benign, malignant, inflammatory, and suspicious) reflects the immediate diagnostic needs in a region where chronic inflammatory diseases like tuberculosis, neglected tropical diseases (lymphatic filariasis, soil-transmitted helminthiases, schistosomiasis, [25]), rhinosporidiosis, actinomyces, or virus-related pathologies can cause organ or tissue enlargement or tumour-like clinical presentations. There is also a need to rule out malignancy in the biopsies of relatively frequent pathological fractures in local children suffering from sickle cell anaemia [6], and malignancy in the endometrium in a district with high prevalence of molar pregnancies [10]. Furthermore, PAP tests and cytology are usually assessed in terms of the diagnostic categories we used [12].

The constrained nature of diagnostic categories we used and the exploratory nature of the study also affect its comparability with other comparative reviews of surgical pathology laboratories. Nevertheless, our result of 13% discordant cytological diagnoses with high hypothetical clinical implications is in the range of major discordances found by Kuijpers et al. (9.1 to 19.4%) in their study of a routine review of fluid/FNA samples by expert cytopathologists. In that study, concordance was at 60.10%, while in our survey it was 56.52%. Furthermore, the proportions of discordance with high hypothetical clinical implications, assuming that our definition overlaps the concept of major error in surgical pathology described by Frable [9], in general adult (5.6%), paediatric/adolescent (2.9%), and lymphoproliferative (6.9%) pathological diagnoses were somewhat higher than those reported in the review by Frable [9], in which the reported rates of major errors in all anatomical sites ranged from 0.26 to 5.7%.

Despite of these limitations, and taking into account that our aim was to evaluate basic categorical discordance, we chose to carry out a much less expensive survey instead of performing the exhaustive process of evaluating pathology misclassifications. Again, it is important to note that our observers live on two different continents, including one in a poor-resource environment. Therefore, we think that our findings are of great value and certainly could be used as a reference point for future analyses. We believe that one of the key factors behind the high overall diagnostic concordance we found was the long period of training at the BMC, during which local personnel had the chance to work (and learn) closely with experienced professionals.

## Conclusion

In conclusion, this blind review on the concordance between pathological and cytological diagnoses from a country with a very high HDI and another with low HDI seemed to be a sensitive and effective method for identifying areas of potential error in cytology and surgical pathology according to Renshaw et al. [20, 21]. This study shows the ability of local pathologists to compare their findings with those of other reviewers, resulting in an acceptable reproducibility with some limitations in cytology and PAP tests, and represents a reference point that can contribute to future, more detailed processes of quality control or audits, be they at the BMC or at other surgical pathology services in limited-resource regions. We think that pathology departments in such settings deserve to be encouraged and rewarded by the international scientific community. Moreover, national governments need to allocate specific funds to improve these laboratories and to establish a stronger commitment to their personnel so they can pursue continuing education programmes, learn ancillary techniques, and optimise their supply of consumables and their equipment maintenance.

## Abbreviations

BL: Burkitt lymphoma
BMC: Bugando Medical Centre
FNA: Fine needle aspiration
HDI: Human development index
NGO: Non-governmental organisation
